# Metabolic networks in a porcine model of trauma and hemorrhagic shock demonstrate different control mechanism with carbohydrate pre-feed

**DOI:** 10.1186/s12873-015-0038-1

**Published:** 2015-07-01

**Authors:** Elizabeth R. Lusczek, Tyrone Vincent, Daniel Lexcen, Vishwesh Kulkarni, Kristine Mulier, Greg Beilman

**Affiliations:** Department of Surgery, University of Minnesota, 516 Delaware St. SE, Minneapolis, MN 55455 USA; Department of Electrical Engineering and Computer Science, Colorado School of Mines, 1610 Illinois St., Golden, CO 80401 USA; Medtronic Inc., 710 Medtronic Parkway NE, Minneapolis, MN 55432 USA; University of Warwick School of Engineering, CV4 7AL Coventry, UK

**Keywords:** Metabolomics, Networks, Hemorrhagic shock

## Abstract

**Background:**

Treatment with oral carbohydrate prior to trauma and hemorrhage confers a survival benefit in small animal models. The impact of fed states on survival in traumatically injured humans is unknown. This work uses regulatory networks to examine the effect of carbohydrate pre-feeding on metabolic response to polytrauma and hemorrhagic shock in a clinically-relevant large animal model.

**Methods:**

Male Yorkshire pigs were fasted overnight (*n* = 64). Pre-fed animals (*n* = 32) received an oral bolus of Karo\textregistered\syrup before sedation. All animals underwent a standardized trauma, hemorrhage, and resuscitation protocol. Serum samples were obtained at set timepoints. Proton NMR was used to identify and quantify serum metabolites. Metabolic regulatory networks were constructed from metabolite concentrations and rates of change in those concentrations to identify controlled nodes and controlling nodes of the network.

**Results:**

Oral carbohydrate pre-treatment was not associated with survival benefit. Six metabolites were identified as controlled nodes in both groups: adenosine, cytidine, glycerol, hypoxanthine, lactate, and uridine. Distinct groups of controlling nodes were associated with controlled nodes; however, the composition of these groups depended on feeding status.

**Conclusions:**

A common metabolic output, typically associated with injury and hypoxia, results from trauma and hemorrhagic shock. However, this output is directed by different metabolic inputs depending upon the feeding status of the subject. Nodes of the network that are related to mortality can potentially be manipulated for therapeutic effect; however, these nodes differ depending upon feeding status.

**Electronic supplementary material:**

The online version of this article (doi:10.1186/s12873-015-0038-1) contains supplementary material, which is available to authorized users.

## Background

Hemorrhagic shock, often a result of traumatic injury, is defined by inadequate tissue perfusion and diminished oxygen delivery with profound consequences for the maintenance of homeostasis. The disruptions to oxygen delivery initiate a switch from aerobic metabolism to anaerobic metabolism as well as a host of compensatory mechanisms in an attempt to preserve homeostasis. Hyperglycemia is a natural consequence of hemorrhage that provides substrate to fuel these mechanisms [1]. Previous work in small animal models has demonstrated the benefit of oral carbohydrate in hemorrhagic shock in providing substrate for the maintenance of homeostasis [2, 3]. A simple oral carbohydrate pre-feed, if beneficial to humans as in small animals, could be an effective prophylactic for at-risk military personnel. The problem of hemorrhagic shock and traumatic injury is complicated in combat settings. Patient care is applied in the field with limited resources and under non-optimal conditions, where 87 % of patients have no documentation of pre-hospital treatment and more than 80 % of deaths due to battlefield trauma are related hemorrhage [4].

This study uses a large animal model to assess the benefit of carbohydrate pre-feed and its associated mechanisms in traumatic injury and hemorrhagic shock. The metabolic component of the response to hemorrhagic shock and traumatic injury is examined here in a model that is designed to (1) assess the metabolic effect of feeding status on hemorrhagic shock, traumatic injury, and resuscitation in large mammals, (2) to reflect battlefield conditions with limited resources for the first hour of resuscitation, and (3) to provide information about control of nodes in the metabolic regulatory network under these conditions. Metabolomics techniques were used to construct serum metabolic profiles for each animal and networks were constructed from these profiles.

## Methods

### Animal preparation and hemorrhagic shock protocol

The animal protocol has been discussed previously [5, 6]. The experimental protocol was approved by the University of Minnesota Animal Use Committee and was conducted in accordance with established guidelines for the ethical and humane treatment of laboratory animals. A well-established model of porcine hemorrhagic shock which has been previously described [7, 8] was utilized (Instrumentation details are presented in the Additional file 1).

Sixty-four male Yorkshire pigs (Manthei Hog Farm, LLC, Elk River, MN) weighing 15–20 kg were randomized to experimental group (*n* = 32, pre-fed and *n* = 32, fasted). The sample size was dictated by power calculations for statistical significance to be observed for multiple variables (e.g. metabolites) at multiple timepoints (baseline, shock, resuscitation). Calculations were done using Statistical Sample Size Calculator PASS 2005. A power of 80 % and type 1 error of 5 % were assumed.

The pre-fed group received a bolus of Karo® Syrup at 7 cc/kg with an equal amount water 30 min prior to experimental manipulation while the fasted group received only water for 12 h prior to experimental manipulation. This dosing was based upon previous work [9]. NMR analysis of Karo® Syrup revealed its components as glucose, fructose, maltose, and sucrose. Serum samples from all animals were taken at set timepoints throughout the experiment (see Table 1). A total of 407 samples were obtained.

**Table 1:** Experimental timeline of serum sample collection and classification into phase of care

	Baseline	Shock 45	Time in hours after shock				
			3	5	9	17	21
Timepoint	B	S45	FR2	FR4	FR8	FR16	FR20
Phase of care	Pre-shock	Shock	Early Resus		Late Resus		

Timepoint designations are used in labeling graphics throughout. The FR label designates full resuscitation; for instance, the FR8 label indicates 8 h of full resuscitation have been administered. Serum sampled at the S45 time point is taken after 45 min of shock

*Resus* Resuscitation

### Experimental trauma and shock

**Fig. 1:**
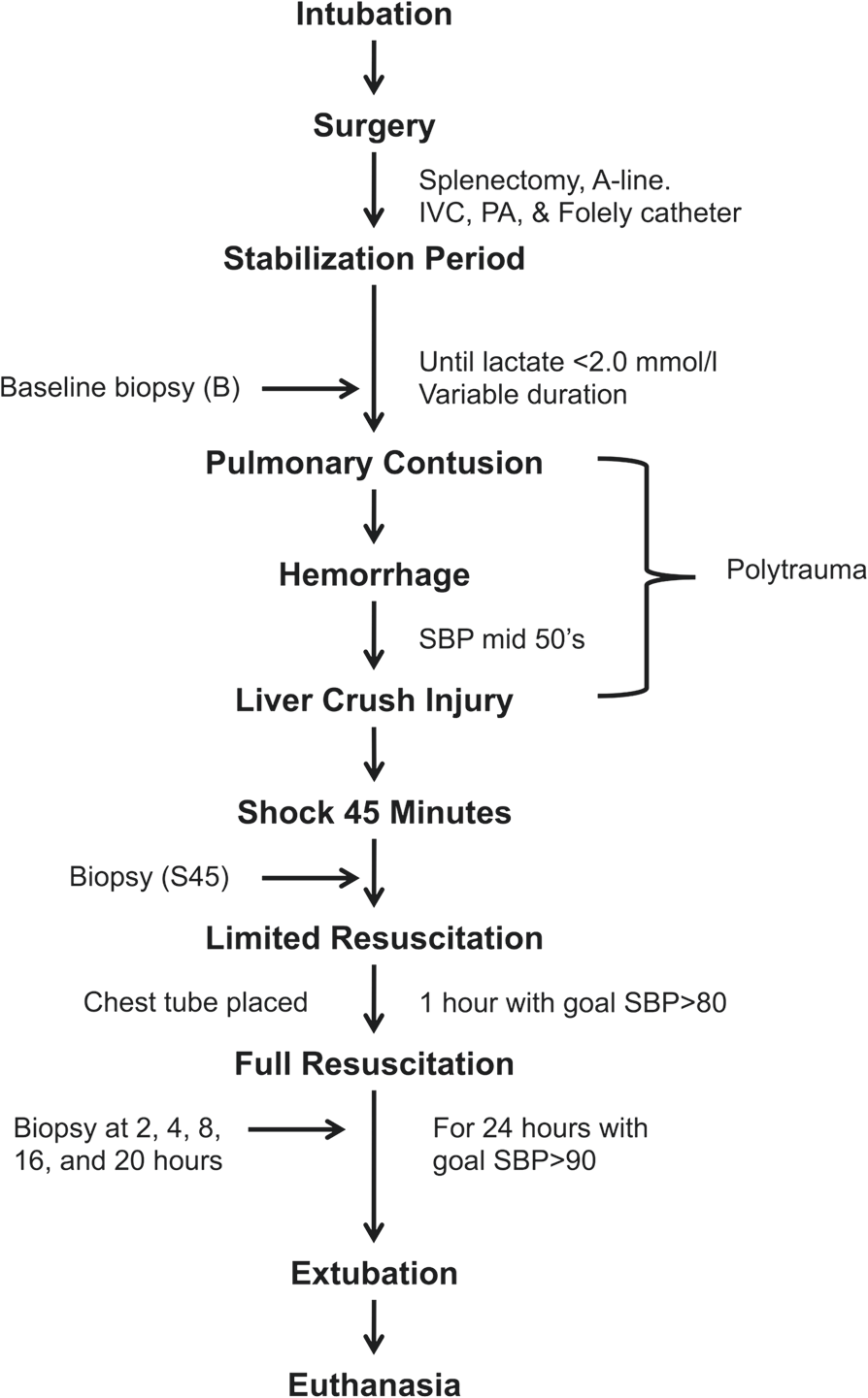
Experimental Timeline. Graphical representation of experimental timeline. Experimental procedures and serum sampling timepoints are shown. Descriptions of sampling timepoints are given in Table 1

For a timeline of the experimental protocol, please see Fig. 1. A captive bolt device was used to create a blunt percussive injury to the right chest. Hemorrhagic shock was then induced by withdrawal of blood from the inferior vena cava until a systolic pressure in the lower 50’s was reached (typically 35 % of total blood volume). Shed blood was placed in an acid-citrate-dextrose bag for later use. A liver crush injury was induced using a Holcomb clamp technique [10], with two crush injuries created in the liver parenchyma. The animal was left in shock for a period of 45 min prior to beginning resuscitation.

### Experimental resuscitation and end of experiment

Animals were resuscitated with lactated Ringer’s fluid given as 20 cc/kg intravenous (IV) boluses to maintain a systolic blood pressure greater than 80 mmHg for 1 h of limited resuscitation, then underwent full resuscitation by algorithm (see Additional file 2). Auto-transfused warmed blood was given at 10 cc/kg IV boluses for a target hemoglobin of greater than 6 g/dL and a urine output of greater than 1 cc/kg/hr. Lactated Ringer's at 20 cc/kg IV boluses or blood at 10 cc/kg/hr were given as needed to maintain blood pressure. After a resuscitation period of 20 h, animals were extubated and sent to recovery. At 24 h after the end of resuscitation, animals were sedated and serum samples were obtained. At 48 h after resuscitation, animals were sedated for endpoint sample harvesting and then euthanized with Beuthanasia D (1 ml/10 kg IV).

### Identification of metabolites from NMR

Harvested blood samples were immediately centrifuged for 15 min at 3000 g to extract serum and were stored at −80 °C until prepared for Nuclear Magnetic Resonance spectroscopy (NMR) analysis. Stored serum was thawed at the time of analysis and internal standard (1 mM trimethylsilylpropionic acid in D_2_O) was added to 500 μL of serum. Samples were transferred into 5 mm NMR tubes (Wilmad, LabGlass, USA).

Proton NMR spectra were taken with a Bruker Avance spectrometer with autosampler and 5 mm triple resonance 1H/13C/15 N TXI CryoProbe with Z-gradient, running TopSpin v. 2.16 (Bruker, Bilerica, MA USA) at 700.13 MHz. A 1D CPMG (Carr-Purcell-Meiboom-Gill) pulse sequence was used to collect spectra for each serum sample. The $90^{\circ}$ pulse width was calibrated for each sample, and was generally 12–13 μs. The relaxation time was defined by each sample's 90° pulse width. During the relaxation period, the protein signal was suppressed. The relaxation delay was 2 s, the acquisition time was 3 s, the spectral width was 10 kHz, the total number of data points collected was 63,000, and 128 transients were collected. All spectra were collected at a temperature of 298 K. Line broadening at 0.5 Hz was applied before FFT; autophasing and auto-baseline correction were applied by TopSpin.

Chenomx software [11] was used to identify and quantify a portion of the metabolites present in each sample. Fine manual phasing and baseline corrections and the software's Reference Deconvolution algorithm were applied to each spectrum before targeted profiling of the metabolites was performed. Forty-eight metabolites were quantified in each sample, resulting in a profile containing the concentration of each identified metabolite in millimoles per liter (mM). Chemical shifts of identified metabolites were compared with those available in the Human Metabolome Database to confirm metabolite identities [12, 13].

### Construction of metabolic networks

Common metabolic interactions were investigated for the fasted and fed groups according to the following procedure. The serum samples provided the time trajectory of the metabolite concentrations at sampling points of 45 min, 3, 5, 9, 17, and 21 h after hemorrhage. This time series data was normalized and shifted by the baseline metabolite concentration before hemorrhage. For each metabolite in series, the rate of change for all animals in the group was estimated using a first difference approximation. Metabolites with sufficiently small error were considered to be ‘controlled nodes’. A regularized linear regression was performed to find the best low order predictor of the rate of change for the controlled node as a function of all 48 measured metabolite concentrations. The metabolite concentrations that were associated with non-zero coefficients in the predictor were considered to be causally affecting the controlled node and were identified as ‘controlling nodes’. The prediction was considered significant if the mean prediction error was less than 50 % than the mean magnitude of the rate of change. The details of the regularization problem are given in the Additional file 3.

## Results

Mortality was 28 % (9/32 animals) in the fasted group (FS) and 47 % (15/32 animals) in the carbohydrate pre-fed group (CPF; log-rank *p* = 0.15 [14]). No survival benefit was observed for the CPF group, an observation that contradicts small animal models.

Six controlled nodes were identified by the algorithm described above: adenosine, cytidine, glycerol, hypoxanthine, lactate, and uridine. These nodes were common to both feeding groups. However, the controlling nodes differed by feeding group as shown in Table 2 (FS) and Table 3 (CPF). To illustrate, succinate and O-phosphocholine were identified as controlling nodes for adenosine, cytidine, and uridine in fasted animals. Phenylalanine was identified as a controlling node for adenosine, cytidine, and uridine in fed animals.

**Table 2:** Controlled nodes and controlling nodes for the FS (fasted) group

Controlled node	Controlling node (sign)
Adenosine	Succinate (−); OPC (+)
Cytidine	Succinate (−); OPC (+); Lactate (−); Adenosine (−)
Glycerol	Uridine (+); Lactate (−)
Hypoxanthine	Succinate (−)
Lactate	BHB (−); OPC (+); Citrate (−); Creatine (−); Isobutyrate (−)
Uridine	Succinate (−); OPC (+); Glutamine (+)

The sign indicates the direction of the interaction relative to the controlled node. (+) indicates that an increase in the level of the controlling node will lead to an increase in the controlled node; (−) indicates that an increase in the level of the controlling node will lead to a decrease in the controlled node

*BHB* 3-hydroxybutyrate, *OPC* O-phosphocholine

**Table 3:** Controlled nodes and controlling nodes for the CPF (fed) group

Controlled node	Controlling node (sign)
Adenosine	PAA (−); AHB (+); Lactate (−)
Cytidine	PAA (−); AHB (+); Formate (−)
Glycerol	Citrate (−); Acetoacetate (+); Lactate (−)
Hypoxanthine	Alanine (−); Glutamate (+)
Lactate	Alanine (−); Glutamate (+); DMA (+)
Uridine	PAA (−); Phenylacetate (+)

The sign indicates the direction of the interaction relative to the controlled node. (+) indicates that an increase in the level of the controlling node will lead to an increase in the controlled node; (−) indicates that an increase in the level of the controlling node will lead to a decrease in the controlled node

*PAA* phenylalanine, *AHB* 2-hydroxybutyrate, *DMA* dimethylamine

**Fig. 2:**
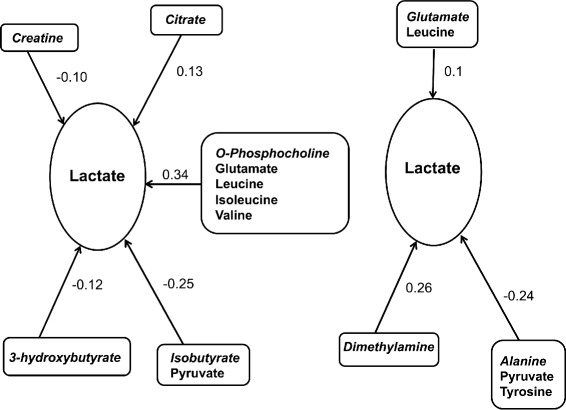
Metabolite Interactions for Lactate. Interactions for the controlled node lactate in FS animals (left) and CPF animals (right). The controlled node lactate is shown in the ovals. Controlling nodes and their correlated signals are shown in boxes, with the controlling node in italics. Interaction weights between the controlled and controlling nodes are shown as arrows and numbers. Arrows are always directed from the controlling nodes to the controlled node to indicate that controlling nodes affect controlled nodes. The sign on the interaction weight associated with each arrow indicates the nature of the relationship. For positive interactions, an increase in the controlling node leads to an increase in the controlled node. For negative interactions, an increase in the controlling node leads to a decrease in the controlled node

**Fig. 3:**
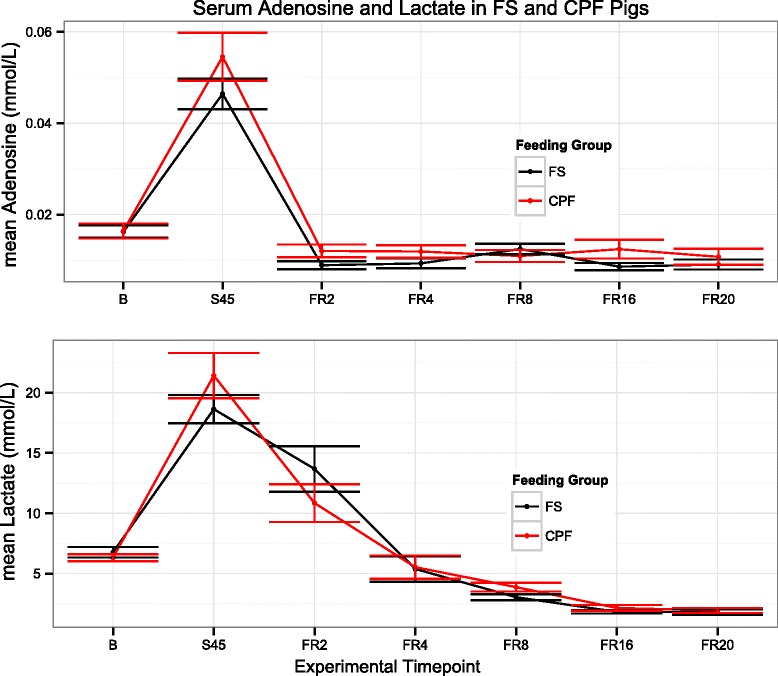
Adenosine and Lactate concentrations. Plotted means and standard errors of Adenosine (top) and Lactate (bottom) concentrations in fasted (FS, shown in black) and carbohydrate pre-fed (CPF, shown in red) pigs

Lactate had the distinction of being both a controlled node and a controlling node for fasted and fed pigs, while adenosine and uridine were both controlled and controlling nodes for fasted pigs only. This indicates a feedback mechanism. A diagram illustrating the interactions for lactate is shown in Fig. 2. Plots of the concentrations of adenosine and lactate are shown in Fig. 3.

Highly correlated signals (R^2^ > 0.8) are presented in Table 4 (FS group) and Table 5 (CPF group). These signals also differed between the two feeding groups. For instance, lactate is correlated with choline and pyruvate in the FS group but with succinate in the CPF group. Distinct modules of controlling nodes and correlated signals are observed to be repeatedly associated with the controlled nodes; however, the composition of these modules depends on whether the subject is fed or fasted. A module consisting of the controlling node O-phosphocholine with correlated signals glutamate, isoleucine, leucine, and valine was associated with controlled nodes adenosine, cytidine, lactate, and uridine in the FS group. A different module, consisting of controlling node phenylalanine with correlated signal tyrosine, was associated with adenosine, cytidine, and uridine in the CPF group.

**Table 4:** Signals highly correlated (R^2^ > 0.8) to the controlling nodes for the FS (fasted) group

Controlling node	Other correlated signals
OPC	Glutamate, Isoleucine, Leucine, Valine
Lactate	Choline, Pyruvate
Isobutyrate	Pyruvate
Glutamine	3-hydroxyisovalerate, Glutamate, Glycine, Histidine, Isoleucine, Leucine, Methionine, Serine, Valine

*OPC* O-Phosphocholine

## Discussion

The serum metabolic regulatory networks presented in this manuscript demonstrate clear differences in the response to trauma, hemorrhagic shock, and resuscitation according to whether pigs were fasted or fed carbohydrate before injury. The analysis presented here is useful in identifying metabolites that could potentially be manipulated for therapeutic effect; these metabolites were identified in Tables 2 and 3. According to the results presented here, the controlled nodes are subject to different mechanisms of control depending upon nutritional status prior to injury. Related work from this study further demonstrates the differences that occur in the response to trauma and hemorrhagic shock as a result of carbohydrate pre-feeding in metabolomics data from other compartments [5, 6] as well as with a wide range of physiologic data [14, 15].

**Table 5:** Signals highly correlated (R^2^ > 0.8) to the controlling nodes for the CPF (pre-fed) group

Controlling node	Other correlated signals
PAA	Tyrosine
Lactate	Succinate
Alanine	Pyruvate, Tyrosine
Glutamate	Leucine

*PAA* Phenylalanine

### Feedback mechanisms and oxidative stress

Nodes that were identified as both controlled nodes and controlling nodes, and therefore represent metabolites subject to a feedback mechanism, are lactate (CPF, FS), uridine (FS), and adenosine (FS). Elevated levels of lactate and adenosine have long been associated with trauma, hemorrhage, and ischemia [16, 17]. While neither elevated lactate nor adenosine is reflective of any single etiology, both are intimately connected with hypoxia and oxidative stress. Uridine has been studied much less by comparison, though it has been shown to accumulate under ischemic conditions in the heart [18]. Differences in oxidative stress were observed between FS and CPF animals [14]. Despite a similar degree of injury, CPF pigs had a significantly lower venous oxygen saturation than FS pigs during shock and resuscitation (*p* < 0.043) and a higher oxygen extraction ratio during resuscitation (*p* = 0.027, see Additional file 4: Table S1).

The behavior of adenosine, a known marker of inflammation and injury [19], may explain observed differences in oxidative stress. Increased levels of adenosine are observed when there is an imbalance in the tissue oxygen supply/demand ratio as occurs during hemorrhagic shock. Activation of adenosine receptors can attenuate the response to ischemia/reperfusion injury and have been exploited for pharmacological pre-conditioning and post-conditioning [20]. For instance, binding of adenosine to A2A receptors of the vasculature induces vasodilation to promote oxygen delivery [21]. Differences in venous oxygen saturation and oxygen extraction ratios between the two groups suggests a greater imbalance in the oxygen supply/demand ratio in CPF animals during shock than in FS animals. The presence of this imbalance between the feeding groups may explain why adenosine displays feedback behavior in FS animals and not CPF animals.

High lactate levels are a standard clinical marker of injury since increases in lactate are indicative of hypoxia. Lactate levels are commonly used as an endpoint for resuscitation and a surrogate of oxygen debt, albeit an imperfect one [22]. High lactate levels are associated with poor outcomes and effective clearance of lactate, facilitated by administration of fluids, blood products, and vasopressors in the clinic, is associated with positive outcomes [1, 23, 24]. Lactate levels between CPF and FS animals were not significantly different, which corroborates lactate's status as (1) an imperfect surrogate of oxygen debt, and (2) a node displaying feedback behavior in both groups.

### Therapeutic targeting of network nodes

Our group has previously reported on the metabolites identified by the network analysis [13]. Succinate and O-phosphocholine were identified by partial least squares discriminant analysis (PLS-DA) as contributing most to differences between fasted animals that lived and fasted animals that died after the shock time point. A similar unpublished analysis of fed animals identified controlling nodes 2-hydroxybutyrate, dimethylamine, glutamate, acetoacetate, and formate as contributing to differences between fed animals that lived and fed animals that died after shock. PLS-DA identified succinate as the most influential metabolite in the survival profile of fed animals, though it was not identified as a node in the network analysis of fed animals. Feedback-associated metabolites lactate and adenosine were found to be strongly associated with hemorrhage in PLS-DA analyses of both fed and fasted animals.

The controlling nodes identified as contributing to survival in previous analyses with PLS-DA are likely nodes to target for therapy. For the FS group, these targets are succinate and O-phosphocholine. For the CPF group, these targets are 2-hydroxybutyrate, acetoacetate, formate, and dimenthylamine. That potential target nodes differ according to feeding status suggests different mechanisms underlie the metabolic response to shock as it relates to mortality. The nodes subject to feedback mechanisms should also be considered for therapeutic purposes, though lactate and adenosine have already been well studied in the context of hemorrhagic shock. Uridine deserves further study.

There were several limitations to this study. The stress of instrumentation and surgical preparation likely influenced the metabolomic and physiologic data. Future work should include sampling at pre-instrumentation timepoints to reflect this. Difficulties in the analysis include the relatively long time between serum samples, which makes estimation of the rate of change difficult, and the strong correlations between metabolite concentration time trajectories. While the effect of the sampling rate could not be avoided, a study of highly correlated concentrations (R^2^ > 0.8) was also made, as potential metabolites in a proposed metabolic network could be replaced with a highly correlated alternate with little loss of predictive power. The identified nodes need to be verified. There is high biologic variation among samples, though the relatively large sample size (32 animals per group) minimizes this to some extent. Finally, the utility of this study is limited under conditions that are not well controlled. It would be possible to feed a soldier a beneficial diet before a mission, but civilian trauma occurs with widely varying intakes of different kinds of food.

## Conclusions

In this study, a common metabolic output that results from trauma, hemorrhagic shock, and resuscitation was observed in regulatory metabolic networks. This output includes adenosine and lactate which are typically associated with oxidative stress. However, the output is directed by drastically different inputs depending upon the feeding status of the subject, suggesting that different mechanisms underlie the metabolic response to trauma depending upon feeding status. Nodes which should be investigated further for targeted therapy include succinate and O-phosphocholine under fasting conditions and glutamate, 2-hydroxybutyrate, acetoacetate, formate, and dimethylamine under carbohydrate-fed conditions.

## Additional files

Additional file 1:
**Instrumentation and Surgical Preparation.** Detailed description of instrumentation and surgical preparation of pigs for trauma and hemorrhagic shock protocol. Additional file 2: Figure S1. Resuscitation Algorithm. Resuscitation protocol used after 1 h of limited resuscitation. If SBP is observed to be less than 90 mmHg, hemoglobin (Hgb) levels are assessed. If hemoglobin is low, shed blood is given; if not, lactated Ringers are given. Alternately, if SBP is not less than 90 mmHg, urine output (UO) is assessed. If urine output is low, the algorithm proceeds according to hemoglobin levels as described. If urine output is adequate, the animal is observed and reassessed as needed. Additional file 3:
**Metabolic Regulatory Network Analysis.** Detailed mathematical description of network analysis. Additional file 4: Table S1. Representative Physiologic Variables. Table of averages and p-values of select physiologic variables. Comparisons were made for statistical significance using Mann–Whitney U tests as described in reference [14]. Statistically significant differences are highlighted in bold text. For further details on physiologic differences between FS and CPF animals, see [14].
