# Supplementary material for: Metabolic networks in a porcine model of trauma and hemorrhagic shock demonstrate different control mechanism with carbohydrate pre-feed
Source: BMC Emerg Med. 2015 Jul 1;15:13. doi: 10.1186/s12873-015-0038-1 (PMC4486709; doi:10.1186/s12873-015-0038-1)
Supplement: Additional file 1: — Instrumentation and Surgical Preparation. Detailed description of instrumentation and surgical preparation of pigs for trauma and hemorrhagic shock protocol. [file 12873_2015_38_MOESM1_ESM.pdf]

SUPPLEMENTARY MATERIAL

1. Instrumentation and Surgical Preparation

All sixty-four animals were anesthetized with an intramuscular dose of telazol (Wyeth Animal Health, Madison, NJ). Anesthesia was maintained by an IV infusion of propofol (2-9 mg/kg, AstraZeneca Pharmaceuticals, Wilmington, England) and 60% inhaled nitrous oxide. Upon sedation, the pigs were orally intubated and ventilated to maintain a partial pressure of oxygen (PO2) of 70-120 torr and a partial pressure of carbon dioxide (PCO2) of 35-45 torr (SERVO Ventilator 900C, Siemens, Malvern, PA). Peripheral intravenous lines were placed in the surgically exposed right femoral artery and right jugular vein. A catheter was placed in the right femoral artery for continuous measurement of blood pressure and blood sampling. An introducer (7 French Avanti, Cordis Corporation, Miami Lakes, FL) was placed into the right jugular vein and a Swan-Ganz catheter (5 French, Edwards Lifesciences, Irvine, CA) was placed for measurements of pulmonary artery pressure, pulmonary wedge pressure, cardiac output, and mixed venous blood sampling. Animals then underwent a midline laparotomy and splenectomy. A Foley catheter was placed in the urinary bladder via stab cystostomy for collection of urine. The inferior vena cava (IVC) was cannulated for blood removal. After surgical preparation, animals were allowed to stabilize until plasma lactate levels reached a value of 2.0 mmol/L or less.
