# Supplementary material for: Metabolic networks in a porcine model of trauma and hemorrhagic shock demonstrate different control mechanism with carbohydrate pre-feed
Source: BMC Emerg Med. 2015 Jul 1;15:13. doi: 10.1186/s12873-015-0038-1 (PMC4486709; doi:10.1186/s12873-015-0038-1)
Supplement: Additional file 2: Figure S1. — Resuscitation Algorithm. Resuscitation protocol used after 1 h of limited resuscitation. If SBP is observed to be less than 90 mmHg, hemoglobin (Hgb) levels are assessed. If hemoglobin is low, shed blood is given; if not, lactated Ringers are given. Alternately, if SBP is not less than 90 mmHg, urine output (UO) is assessed. If urine output is low, the algorithm proceeds according to hemoglobin levels as described. If urine output is adequate, the animal is observed and reassessed as needed. [file 12873_2015_38_MOESM2_ESM.pdf]

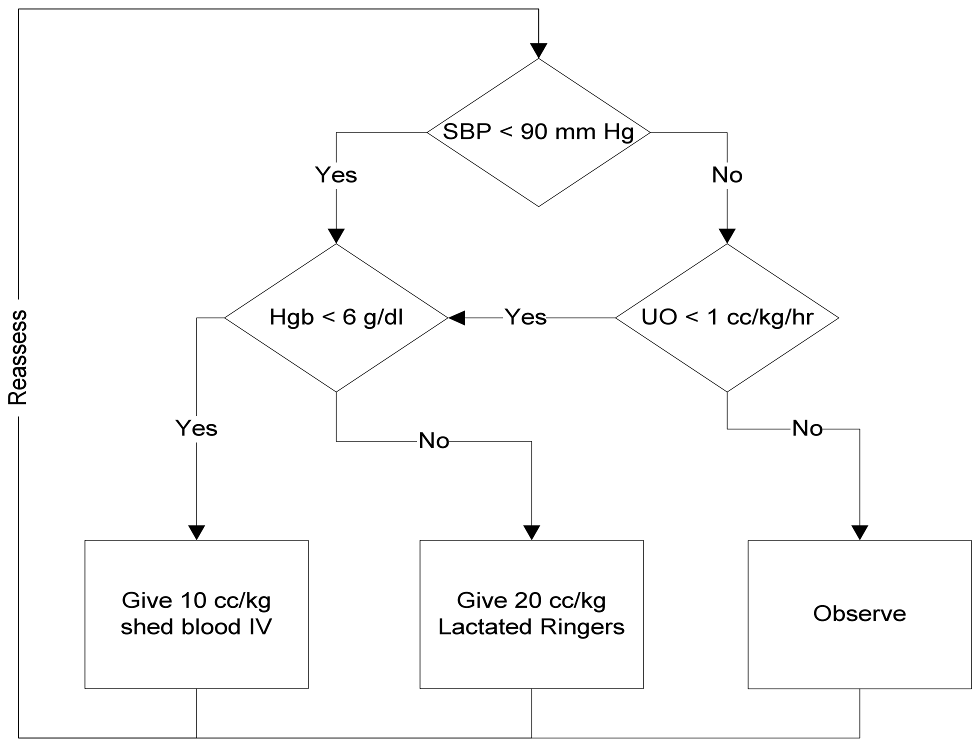

Supplemental Figure 1: Resuscitation algorithm used for shocked, resuscitated animals (Fasted and Pre-fed groups). If SBP is observed to be less than 90 mmHg, hemoglobin (Hgb) levels are assessed. If hemoglobin is low, shed blood is given; if not, lactated Ringers are given. Alternately, if SBP is not less than 90 mmHg, urine output (UO) is assessed. If urine output is low, the algorithm proceeds according to hemoglobin levels as described. If urine output is adequate, the animal is observed and reassessed for the full resuscitation period.
