# Supplementary material for: Metabolic networks in a porcine model of trauma and hemorrhagic shock demonstrate different control mechanism with carbohydrate pre-feed
Source: BMC Emerg Med. 2015 Jul 1;15:13. doi: 10.1186/s12873-015-0038-1 (PMC4486709; doi:10.1186/s12873-015-0038-1)
Supplement: Additional file 3: — Metabolic Regulatory Network Analysis. Detailed mathematical description of network analysis. [file 12873_2015_38_MOESM3_ESM.pdf]

SUPPLEMENTARY MATERIAL

1. Metabolite network analysis

The network analysis is based on the method of [1]. The following data analysis is performed separately for the fasted group and the fed group. Each set of data contains 48 metabolites measured at at a baseline, and 6 sampling times, measured in hours after hemorrhage. The baseline is denoted time $t_0 = 0$, and the other sampling times are $\{t_k\}_{k=1}^{6} = \{0.75, 3, 5, 9, 17, 21\}$ as described in Table 1 of the manuscript.

- Let $x_\ell(k)$ be the data for Metabolite ℓ at sample time t_k.
- Pre-processing:
  - The data is normalized as follows: For all ℓ, set
  $$\bar{x}_\ell(k) = \frac{x_\ell(k)}{\sqrt{\sum_{k=0}^{6} x_\ell^2(k)}}$$
  - The data is shifted so that the baseline measurement is 0 as follows:
  $$\tilde{x}_\ell(k) = \bar{x}_\ell(k) - \bar{x}_\ell(0)$$
- Regression Matrix: The data from all metabolites is placed into a matrix, with each column a different metabolite, and each row a different sampling time, starting after hemorrhage and continuing until the 5th sample.
$$A = \begin{bmatrix} \tilde{x}_1(1) & \tilde{x}_2(1) & \cdots & \tilde{x}_{48}(1) \\ \tilde{x}_1(2) & \tilde{x}_2(2) & \cdots & \tilde{x}_{48}(2) \\ \vdots & \vdots & \ddots & \vdots \\ \tilde{x}_1(5) & \tilde{x}_2(5) & \cdots & \tilde{x}_{48}(5) \end{bmatrix}$$
- For $n = 1$ to 48:
  - The metabolite n is chosen as the "controlled node". The data that is to be predicted is the change in this metabolite from one sample to the next. This is denoted by $\Delta\tilde{x}_n(k)$, where $\Delta\tilde{x}_n(k) = (\tilde{x}_n(k+1) - \tilde{x}_n(k))/(t_{k+1} - t_k)$. Since there are 6 samples, we can calculate $\Delta\tilde{x}_n(k)$ for $k = 1, \cdots, 5$. Set
  $$y = \begin{bmatrix} \Delta\tilde{x}_n(1) \\ \Delta\tilde{x}_n(2) \\ \vdots \\ \Delta\tilde{x}_n(5) \end{bmatrix}$$
  - The following re-weighted regularized optimization is used to select regressors and weights that best explain the rate of change of the metabolite concentration of the controlled node. The regularization $\|Wz\|_1$ promotes sparsity in the solution, so that only a few elements of z will be non-zero. Select $t = .95$, $\delta = .001$ and length 48 vector w as $w_i = 1$ for $i = 1, \cdots, 48$. For j=1 to 3, do the following
    - Set $W = \text{diag}(w)$,
    $$\begin{array}{cc} \min_{z,\epsilon} & t\|Wz\|_1 + (1-t)\epsilon \\ \text{subject to} & \|y - Ax\|_2 \le \epsilon \end{array}$$
    where for length m vector x, $\|x\|_2 = \sqrt{\sum_{i=1}^{m} x_i^2}$, and $\|x\|_1 = \sum_{i=1}^{m} |x_i|$.
    - set
    $$w(i) = \frac{\delta}{\delta + |z(i)|}$$
  - Determine if $\epsilon < \frac{1}{2}(\|y_{i_1}\|_2 + \|y_{i_2}\|_2 + \cdots + \|y_{i_m}\|_2)$. If so, this is noted as a potentially interesting node. Find the indices i for which the weights $|z(i)| > 0.1$ and denote the metabolites i as controlling node n.

References

[1] Zavlanos, M.M., Julius, A.A., Boyd, S.P., Pappas, G.J.: Inferring stable genetic networks from steady-state data. Automatica **47**(6), 1113–1122 (2011)
