# Supplementary material for: Metabolic networks in a porcine model of trauma and hemorrhagic shock demonstrate different control mechanism with carbohydrate pre-feed
Source: BMC Emerg Med. 2015 Jul 1;15:13. doi: 10.1186/s12873-015-0038-1 (PMC4486709; doi:10.1186/s12873-015-0038-1)
Supplement: Additional file 4: Table S1. — Representative Physiologic Variables. Table of averages and p-values of select physiologic variables. Comparisons were made for statistical significance using Mann–Whitney U tests as described in reference [14]. Statistically significant differences are highlighted in bold text. For further details on physiologic differences between FS and CPF animals, see [14]. [file 12873_2015_38_MOESM4_ESM.docx]

| **Timepoint** | **Pig Group** | **ERO2** | **p-value** | **VO2 Sat (%)** | **p-value** | **Cardiac Output (L/min)** | **p-value** | **Heart Rate (BPM)** | **p-value** | **BUN (g/dL)** | **p-value** | **Lactate (mmol/L)** | **p-value** | **Glucose (mg/dL)** | **p-value** |
| --- | --- | --- | --- | --- | --- | --- | --- | --- | --- | --- | --- | --- | --- | --- | --- |
| B | FS | 26.08 | 0.33 | 72.63 | 0.28 | **2.57** | **0.03** | 117.66 | 0.63 | **8.00** | **0.00** | 1.33 | 0.78 | **100.03** | **<0.0001** |
| B | CPF | 24.33 |  | 74.59 |  | **2.92** |  | 121.16 |  | **6.31** |  | 1.30 |  | **161.78** |  |
| S45 | FS | 77.82 | 0.15 | **21.07** | **0.04** | 1.02 | 0.53 | 229.54 | 0.59 | **10.34** | **<0.0001** | 6.41 | 0.06 | **123.30** | **<0.0001** |
| S45 | CPF | 81.29 |  | **16.82** |  | 1.09 |  | 226.19 |  | **7.79** |  | 8.03 |  | **331.00** |  |
| FR2 | FS | **42.17** | **0.03** | **55.23** | **0.01** | 2.66 | 0.73 | **154.62** | **0.02** | **11.36** | **0.00** | 4.14 | 0.90 | 103.00 | 0.09 |
| FR2 | CPF | **50.90** |  | **44.09** |  | 2.55 |  | **189.10** |  | **8.52** |  | 4.20 |  | 128.61 |  |
| FR4 | FS | 26.96 | 0.25 | 70.68 | 0.15 | 4.75 | 0.36 | 186.64 | 0.90 | **12.48** | **0.00** | 1.60 | 0.52 | 108.00 | 0.88 |
| FR4 | CPF | 32.19 |  | 64.32 |  | 4.37 |  | 181.82 |  | **9.45** |  | 2.05 |  | 108.14 |  |
| FR8 | FS | 27.52 | 0.63 | 69.88 | 0.47 | 4.34 | 0.44 | 157.28 | 0.17 | **13.48** | **0.00** | 0.78 | 0.27 | 103.71 | 0.12 |
| FR8 | CPF | 30.59 |  | 65.77 |  | 4.41 |  | 164.48 |  | **10.81** |  | 0.99 |  | 115.68 |  |
| FR16 | FS | 24.53 | 0.34 | 73.68 | 0.12 | 4.42 | 0.12 | 146.40 | 0.42 | **13.08** | **0.02** | 0.73 | 0.97 | **75.28** | **0.03** |
| FR16 | CPF | 28.36 |  | 67.05 |  | 3.86 |  | 140.36 |  | **11.09** |  | 0.82 |  | **85.45** |  |
| FR20 | FS | 31.01 | 0.49 | 64.68 | 0.54 | 3.62 | 0.79 | 140.60 | 0.91 | 12.79 | 0.14 | 0.77 | 0.54 | 75.56 | 0.41 |
| FR20 | CPF | 35.54 |  | 59.24 |  | 3.46 |  | 139.75 |  | 11.82 |  | 0.93 |  | 79.38 |  |
